# Urinary soluble (pro)renin receptor excretion is associated with urine pH in humans

**DOI:** 10.1371/journal.pone.0254688

**Published:** 2021-07-26

**Authors:** Nobukazu Sasaki, Satoshi Morimoto, Chikahito Suda, Satoru Shimizu, Atsuhiro Ichihara

**Affiliations:** 1 Department of Endocrinology and Hypertension, Tokyo Women’s Medical University, Tokyo, Japan; 2 School of Arts and Sciences, Tokyo Woman’s Christian University, Tokyo, Japan; International University of Health and Welfare, School of Medicine, JAPAN

## Abstract

The (pro)renin receptor [(P)RR] binds to renin and its precursor prorenin to activate the tissue renin-angiotensin system. It is cleaved to generate soluble (P)RR and M8–9, a residual hydrophobic truncated protein. The (pro)renin receptor also functions as an intracellular accessory protein of vacuolar-type H^+^-ATPase, which plays an essential role in controlling the intracellular vesicular acid environment. Thus, in the kidney, (P)RR may play a role in transporting H^+^ to urine in the collecting duct. Although blood soluble (P)RR has been recognized as a biomarker reflecting the status of the tissue renin-angiotensin system and/or tissue (P)RR, the significance of urinary soluble (P)RR excretion has not been determined. Therefore, this study aimed to investigate the characteristics of urinary soluble (P)RR excretion. Urinary soluble (P)RR excretion was measured, and its association with background factors was investigated in 441 patients. Relationships between changes in urine pH due to vitamin C treatment, which reduce urine pH, and urinary soluble (P)RR excretion were investigated in 10 healthy volunteers. Urinary soluble (P)RR excretion was 1.46 (0.44–2.92) ng/gCre. Urine pH showed a significantly positive association with urinary soluble (P)RR excretion, independent of other factors. Changes in urine pH and urinary soluble (P)RR excretion due to vitamin C treatment were significantly and positively correlated (ρ = 0.8182, p = 0.0038). These data showed an association between urinary soluble (P)RR excretion and urine pH in humans, suggesting that (P)RR in the kidney might play a role in urine pH regulation.

## Introduction

The (pro)renin receptor [(P)RR], which consists of 350 amino acids with a single transmembrane domain and binds to renin and prorenin, is widely expressed in various tissues, including the heart, brain, and kidney [1]. The binding of prorenin to the extracellular domain of the (P)RR induces non-proteolytic renin activation [2], which accelerates the conversion of angiotensinogen to angiotensin. This process plays a key role in regulating the tissue renin-angiotensin system (RAS) [1]. The (P)RR also stimulates its own intracellular signal transduction pathways, such as the mitogen-activated protein kinase (MAPK) pathways, independent of the RAS [1]. (P)RR also functions as an accessory protein of vacuolar H^+^-ATPase (V-ATPase), which is an ATP-dependent proton pump that plays an important role in the transportation of protons across plasma membranes, acidifies the intracellular compartments [3], and plays a role in secretion and membrane fusion independent of acidification [4–7]. The (P)RR also serves as an adaptor protein between the V-ATPase and Wnt receptor complex [8], which is involved in virtually every aspect of embryonic development and in homeostatic self-renewal [9]. In addition, binding of (P)RR to pyruvate dehydrogenase E1 β-subunit (PDHB) prevents PDHB phosphorylation and maintains aerobic glucose metabolism [10]. Therefore, (P)RR is considered a multifunctional protein that exhibits complex structure and functionality [11].

(P)RR is cleaved to be soluble (P)RR [s(P)RR], which is secreted into the extracellular space and is ultimately found in both blood and urine [12, 13]. Blood s(P)RR may serve as a biomarker that reflects the expression levels of tissue RAS and/or tissue (P)RR [14, 15]. However, the significance of urinary s(P)RR excretion in humans remains unclear. Therefore, the present study investigated the characteristics of urinary s(P)RR excretion in humans.

## Materials and methods

### Study 1

This study enrolled 441 consecutive patients who visited the Department of Endocrinology and Hypertension at the Tokyo Women’s Medical University Hospital between September 2013 and February 2016. Power analyses could not be performed because no preliminary data on urinary s(P)RR excretion in humans are available. This study was conducted according to the principles of the 1975 Declaration of Helsinki, as revised in 2013. Patients who had suffered from acute myocardial infarction or stroke in the previous 6 months, pregnant women, and those with apparent peripheral vascular or malignant disease were excluded. All participants were enrolled after obtaining written informed consent and approval from the ethical committee of Tokyo Women’s Medical University (approval #: 2303).

#### Background factors

At enrollment, information was collected on sex, age, hypertension, body mass index (BMI), and waist circumference (WC). Hypertension was diagnosed when the patients were already under treatment with antihypertensive agents or met the criteria of the Japanese Society of Hypertension Guidelines for the Management of Hypertension (JSH 2019) [16]. WC was measured at the level of the umbilicus while the patient was standing and after exhaling.

#### Visceral fat area

Visceral fat area (VFA) was estimated by the bioelectrical impedance analysis method using HDS-2000 DUALSCAN^®^ (Omron Healthcare Co., Kyoto, Japan) in the morning after a 12-h fast, as previously described [17].

#### Blood pressure and pulse rate

Office blood pressure (BP) and pulse rate (PR) were measured with the patient in a sitting position after resting for at least 5 min using an automated sphygmomanometer.

#### Urinary examinations

Spot urine samples were obtained, and the pH and concentrations of albumin (Alb) and creatinine (Cre) were quantified using standardized assessment methods at our clinical laboratory. Urinary serum soluble s(P)RR concentrations were measured using an enzyme-linked immunosorbent assay kit (Takara Bio, Otsu City, Japan), consisting of a solid-phase sandwich enzyme-linked immunosorbent assay with a highly specific antibody [18]. For this measurement, 100 μL of addition agent (0.25% casein-Na, 5% BSA, 0.05% NaN_3_) was added to 10 ml of spot urine to prevent adsorption to the test tube wall. Excretions of Alb or s(P)RR were determined by dividing these values by the Cre concentrations.

#### Blood examinations

Blood samples were collected while patients were in the sitting position after at least 15 min of rest and an overnight fast. Hemoglobin (Hb), uric acid, creatinine, calcium (Ca), inorganic phosphorus (IP), albumin, intact parathyroid hormone (i-PTH), blood sugar (BS), insulin, hemoglobin A1c (HbA1c), low-density lipoprotein (LDL)-cholesterol, high-density lipoprotein (HDL)-cholesterol, triglyceride (TG), high-sensitivity C-reactive protein (hsCRP), and brain natriuretic peptide (BNP) levels were measured using standard laboratory methods at our clinical laboratory. The corrected Ca was calculated using the formula: corrected Ca = Ca–Alb + 4.0 (mg/dl (if Alb < 4.0) [19]. The estimated glomerular filtration rate (eGFR) was calculated using the following equation:

$$\text{eGFR}\;(\text{mL}/\text{min}/1.73{\;\text{m}}^{2})=194\times {\text{Cre}}^{-1.094}\times {\text{age}}^{-0.287}\;(\times 0.739,\;\text{if}\;\text{female})$$

[20]. Serum soluble s(P)RR concentrations were measured using an enzyme-linked immunosorbent assay kit (Takara Bio, Otsu City, Japan) [18].

#### Western blot analysis for s(P)RR using urine samples

Urine was obtained from five patients. Urine, Chinese hamster ovary cells transfected with cDNA for (P)RR as a positive control, and an electrolyte solution (dialysate: LYMPAC TA1; Nipro Corp, Osaka, Japan) as a negative control were lysed using a solution containing 2% SDS, 10% glycerol, 50 mM Tris-HCl (pH 6.8), and 100 mM dithiothreitol, boiled, and subjected to western blot analysis using 93A1B, an anti-(P)RR antibody, supplied by the s(P)RR ELISA kit. The reactivity of the antibodies was assessed by immunoprecipitation using western blotting. Urine samples and positive and negative control samples were incubated with 93A1B, and then Protein G Sepharose (GE Healthcare Japan, Hino City, Japan) was added. After further incubation, the samples were centrifuged, and the resulting pellets were washed three times. The pellets were subsequently lysed, and western blot analysis was performed using 93A1B.

#### Study protocol

The relationship between background factors, VFA, office BP and PR, urinary and blood data, and urinary s(P)RR excretion or serum s(P)RR concentration were examined by single or multiple regression analyses.

#### Statistical analyses

All data are expressed as the mean ± standard deviation or median value (interquartile range). The Mann-Whitney *U* test was used to compare the two groups. A single correlation analysis was performed using Spearman’s rank correlation to determine the association between background factors, VFA, office BP and PR, urinary and blood data, and urinary s(P)RR excretion or serum s(P)RR concentration. Multiple regression analysis was used to identify the determinants of urinary s(P)RR excretion or serum s(P)RR concentration by forward stepwise analysis. The level of significance was set at P < 0.05. All statistical analyses were performed using JMP Pro version 15 (SAS Institute Inc., Cary, NC, USA).

### Study 2

Ten male volunteers without any past medical history were recruited between February and March 2017 among students at Keio University (Tokyo, Japan) and were enrolled after obtaining written informed consent. At enrollment, BP and PR, fasting BS, HbA1c, and serum creatinine levels were measured using the same methods as in ***Study 1***. In addition, a qualitative urine test was performed at enrollment. They were treated with 600 mg of ascorbic acid calcium pantothenate, a vitamin C tablet, thrice daily for 14 days to decrease the urine pH. This study was conducted according to the principles of the 1975 Declaration of Helsinki, as revised in 2013, and was approved by the ethical committee of Tokyo Women’s Medical University (approval #: 170308).

#### Study protocol

Urine pH and urinary s(P)RR excretion were measured in spot urine samples obtained before and after treatment using the same methods as in ***Study 1***. The relationship between changes in urine pH and urinary s(P)RR excretion due to vitamin C treatment was investigated.

#### Statistical analyses

All data are expressed as means ± standard deviations or as medians (interquartile range). Wilcoxon signed-rank tests were applied to determine the difference in urine pH or urinary s(P)RR expression before and after treatment. Single correlation analysis was performed using Spearman’s rank correlation to determine the association between changes in urine pH or urinary s(P)RR excretion due to the treatment. The level of significance was set at P < 0.05. All statistical analyses were performed using JMP Pro version 15 (SAS Institute Inc., Cary, NC, USA).

## Results

### Study 1

#### Characteristics of the study participants

A total of 441 patients (133 patients with essential hypertension, 143 patients with secondary hypertension, and 165 patients with other endocrine or metabolic disorders) were enrolled in this study. Table 1 shows the background factors, VFA, office BP and PR, and urinary and blood data. The median urinary s(P)RR excretion level was 1.46 (interquartile range: 0.44–2.92) ng/gCre and the median serum s(P)RR level was 23.8 (interquartile range: 21.0–27.2) ng/gCre.

**Table 1:** Characteristics of the study subjects.

Sex (male/female)	267/174
Age (y.o.)	54 (44–66)
Hypertension (yes/no)	286/155
BMI (kg/m^2^)	23.8 (21.5–26.4)
Waist circumference (cm)	87.2 (80.0–95.0)
Visceral fat area (cm^2^)	73.0 (48.3–105.8)
Office BP and pulse rate	
Systolic BP (mmHg)	131±20
Diastolic BP (mmHg)	80±15
Pulse rate (beats/min)	69 (61–77)
Urine tests	
pH	6.321 (5.800–6.820)
Log pH	1.84 (1.76–1.92)
Creatinine (mg/dl)	88 (55–135)
Albumin (mg/g creatinine)	7.9 (4.6–20.0)
s(P)RR/creatinine (ng/g creatinine)	1.46 (0.44–2.92)
Log s(P)RR/creatinine	0.38 (-0.82–1.07)
Blood tests	
Hemoglobin (g/dl)	13.5±1.4
Uric acid (mg/dl)	5.1 (4.3–6.0)
Creatinine (mg/dl)	0.69 (0.59–0.83)
eGFR (ml/min/1.73 m^2^)	77.0 (65.5–88.8)
Corrected calcium (mg/dl)	9.0 (8.8–9.2)
Inorganic phosphorus (mg/dl)	3.5 (3.1–3.9)
Albumin (g/dl)	4.2 (3.9–4.4)
Intact–PTH (pg/ml)	52 (40–65)
Blood sugar (mg/dl)	93 (87–102)
Insulin (nmol)	4.9 (3.3–8.0)
Hemoglobin A1c (%)	5.7 (5.5–6.0)
LDL-cholesterol (mg/dl)	118 (97–140)
HDL-cholesterol (mg/dl)	58 (48–70)
Triglyceride (mg/dl)	106 (76–147)
hs CRP (ng/ml)	476 (186–1250)
s(P)RR (ng/ml)	23.8 (21.0–27.2)

Data are presented as the means ± standard deviations and medians (interquartile range). Abbreviations: BMI, body mass index; BP, blood pressure; s(P)RR, soluble (pro)renin receptor; eGFR, estimated glomerular filtration rate; PTH, parathyroid hormone; LDL, low-density lipoprotein; HDL, high-density lipoprotein; hsCRP, high-sensitivity C-reactive protein; BNP, brain natriuretic peptide.

#### Detection of urinary s(P)RR excretion

**Fig 1:**
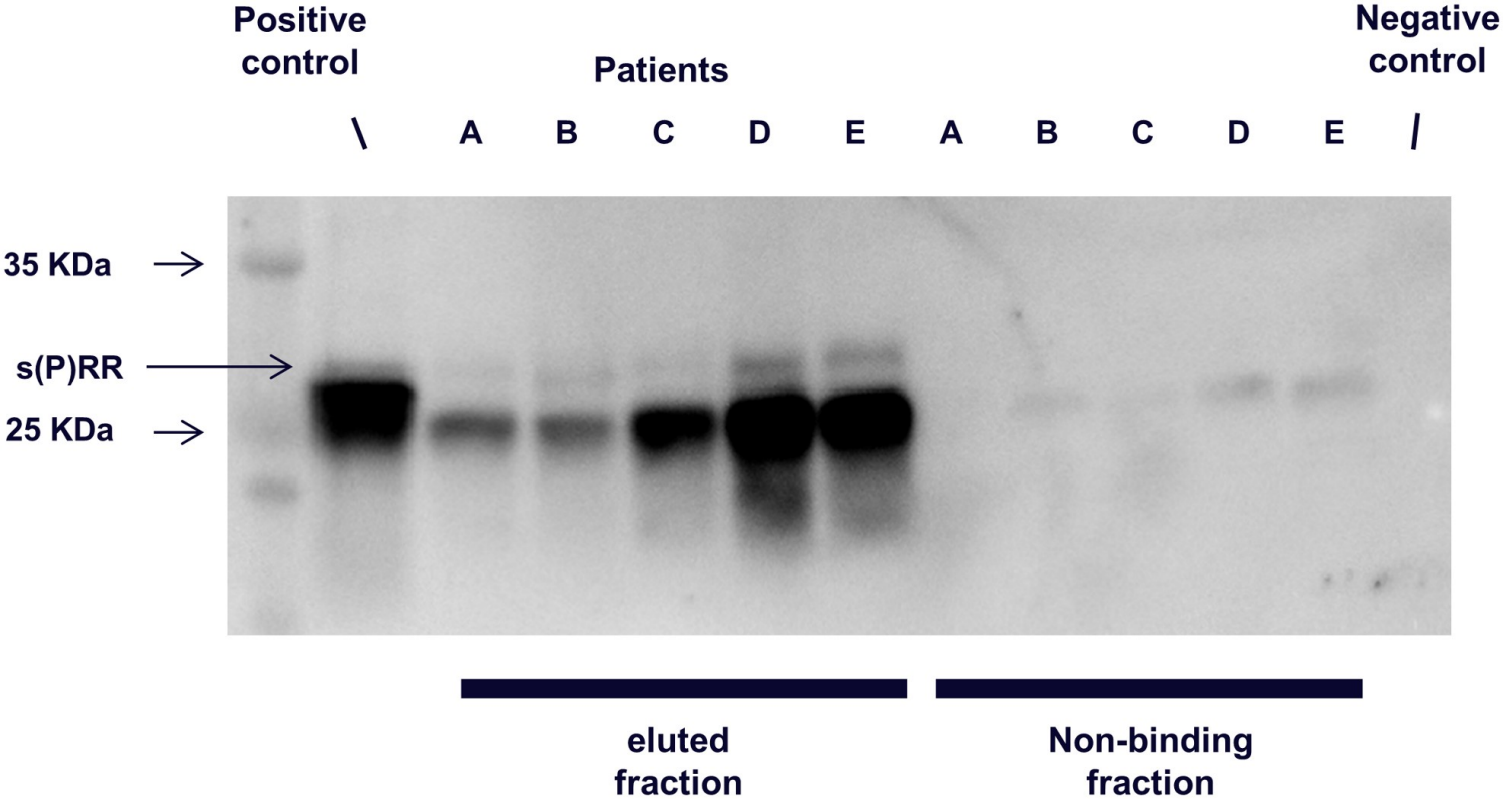
Western blotting and immunoprecipitation analysis. Identification of urinary soluble (Pro) renin receptor in five patients (A, B, C, D, and E). s(P)RR, soluble (Pro) renin receptor.

The results of the immunoprecipitation western blotting are shown in Fig 1. In all five of the urinary s(P)RR samples, a band indicating s(P)RR was detected, confirming that s(P)RR was excreted into the urine.

#### Single correlation analysis with urinary s(P)RR excretion levels

**Fig 2:**
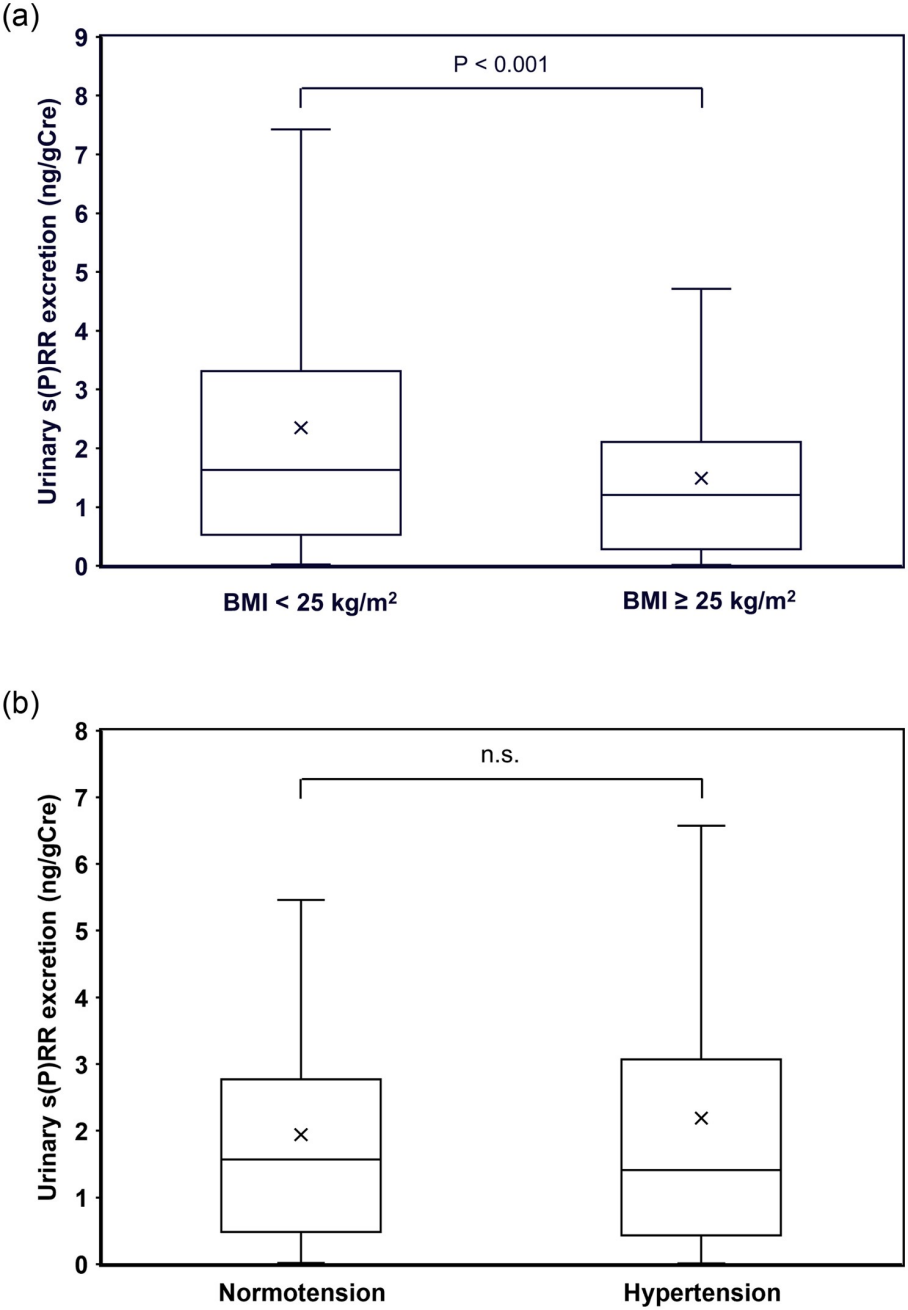
Comparisons of urinary s(P)RR excretion between patients with and without obesity (a) and those with and without hypertension (b). s(P)RR, soluble (pro)renin receptor; BMI, body mass index.

**Fig 3:**
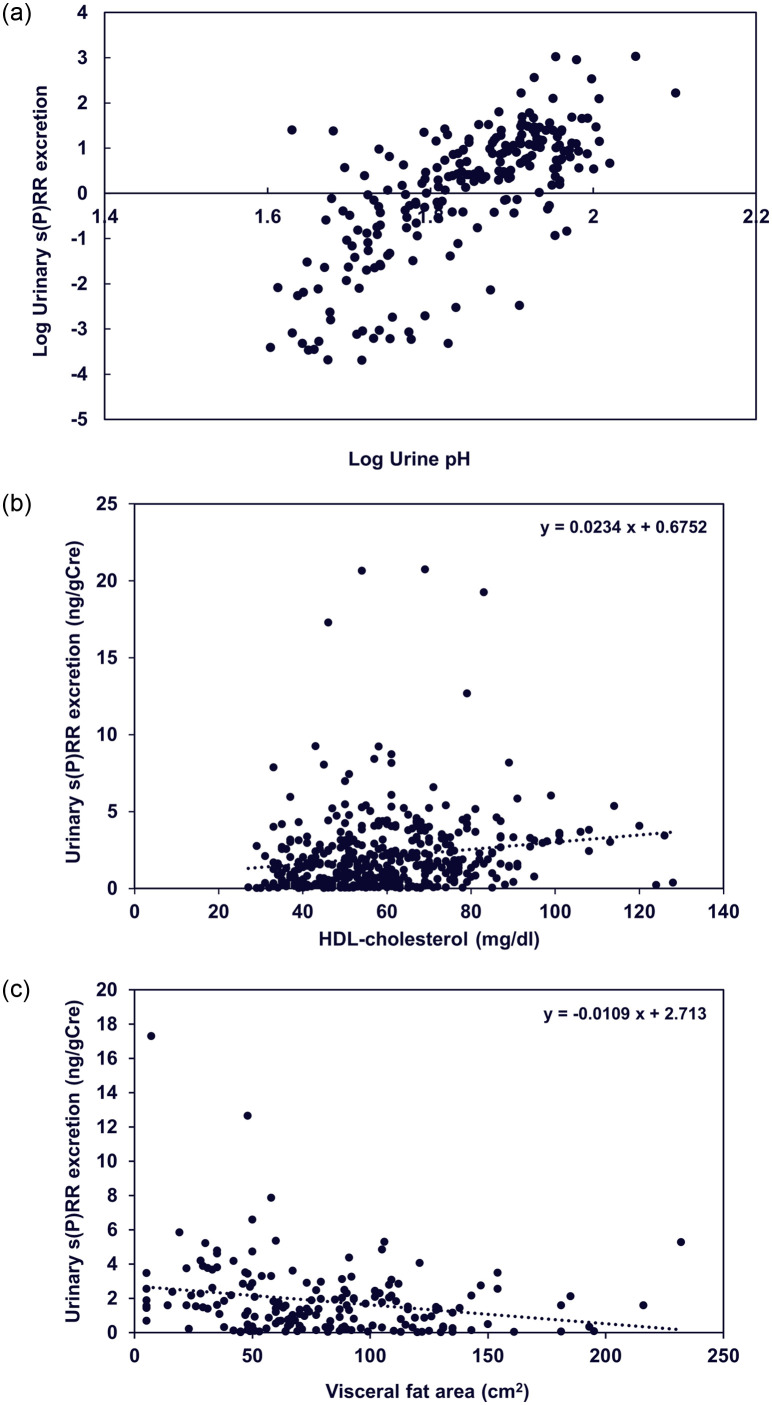
Scattergrams showing relationship between log urine pH (a) and log urinary s(P)RR excretion (a) and HDL-cholesterol (b) or visceral fat area (c) and urinary s(P)RR excretion. s(P)RR, soluble (pro)renin receptor; HDL, high-density lipoprotein.

Urinary s(P)RR excretion was significantly higher in women (1.73, 0.60–3.33 ng/gCre) than in men (1.13, 0.22–2.22 ng/gCre) (P < 0.0001), which was considered to be due to significantly lower urinary Cre concentration in women (73, 46–10 mg/dl) compared to men (109, 77–159 mg/dl) (P < 0.0001), particularly since the urinary s(P)RR concentrations were similar between women (10.4, 5.22–18.66 ng/ml) and men (9.75, 2.87–21.1 ng/ml). Urinary s(P)RR excretion was significantly lower in patients with BMI ≥ 25 kg/m^2^ than in those with BMI < 25 kg/m^2^ (Fig 2a), while it was not significantly different between those with normotensive and hypertensive patients (Fig 2b). In the single correlation analyses, urine pH, eGFR, and HDL-cholesterol were significantly and positively correlated with urinary s(P)RR excretion and log urine pH was significantly as well as positively correlated with log urinary s(P)RR excretion (Table 2, Fig 3a and 3b). BMI, WC, VFA, uric acid, Cre, IP, BS, insulin, TG, hs-CRP, and serum s(P)RR levels were significantly and negatively correlated with urinary s(P)RR excretion levels (Table 2, Fig 3a–3c). Urine pH showed the strongest relationship with urinary s(P)RR excretion levels (Table 2).

**Table 2:** Single correlation analyses with urinary soluble (pro)renin receptor excretion level.

Variables	ρ	P
Age	0.019	0.697
BMI	-0.203	<0.001
Waist circumference	-0.266	<0.001
Visceral fat area	-0.256	<0.001
Office BP and pulse rate		
Systolic BP	-0.006	0.900
Diastolic BP	0.015	0.768
Pulse rate	-0.023	0.676
Urine tests		
pH	0.682	<0.001
Albumin	0.092	0.065
Blood tests		
Hemoglobin	-0.062	0.193
Uric acid	-0.267	<0.001
Creatinine	-0.267	<0.001
eGFR	0.168	<0.001
Corrected calcium	-0.087	0.103
Inorganic phosphorus	-0.108	0.040
Albumin	0.055	0.287
Intact-PTH	0.042	0.541
Blood sugar	-0.135	0.005
Insulin	-0.255	<0.001
Hemoglobin A1c	-0.036	0.456
LDL-cholesterol	0.011	0.819
HDL-cholesterol	0.245	<0.001
Triglyceride	-0.214	<0.001
hs CRP	-0.137	0.005
s(P)RR	-0.162	0.001

Abbreviations: BMI, body mass index; BP, blood pressure; eGFR, estimated glomerular filtration rate; PTH, parathyroid hormone; LDL, low-density lipoprotein; HDL, high-density lipoprotein; hs-CRP, high-sensitivity C-reactive protein; BNP, brain natriuretic peptide; s(P)RR, soluble (pro)renin receptor.

#### Multiple regression analyses with urinary s(P)RR excretion

To compare the strength of the relationship between urinary s(P)RR excretion level and the possible predictor variables, multiple regression analysis testing with urine pH, uric acid, eGFR, TG, serum s(P)RR concentration, and VFA as independent variables was performed. Urine pH and VFA were selected as the independent variables. Urine pH was significantly and positively associated and VFA was significantly and negatively associated with urinary s(P)RR excretion. The association was stronger in urine pH than in VFA, indicating that urine pH has the strongest relationship with urinary s(P)RR excretion, independent of other factors (Table 3a).

**Table 3:** Multiple regression analyses.

a. Multiple regression analysis with urinary soluble(pro)renin receptor excretion.			
Variables	β	P	VIF
Urine pH	0.489	<0.001	1.011
VFA	-0.181	<0.001	1.011
R^2^ = 0.290, P < 0.0001 for entire model			
b. Multiple regression analysis with serum soluble(pro)renin receptor concentration.			
Variable	β	P	
eGFR	-0.214	<0.001	
R^2^ = 0.046, P < 0.0001 for entire model			

Abbreviations: VIF, variance inflation factor; VFA, visceral fat area; eGFR, estimated glomerular filtration rate.

#### Single correlation analysis with serum s(P)RR levels

**Fig 4:**
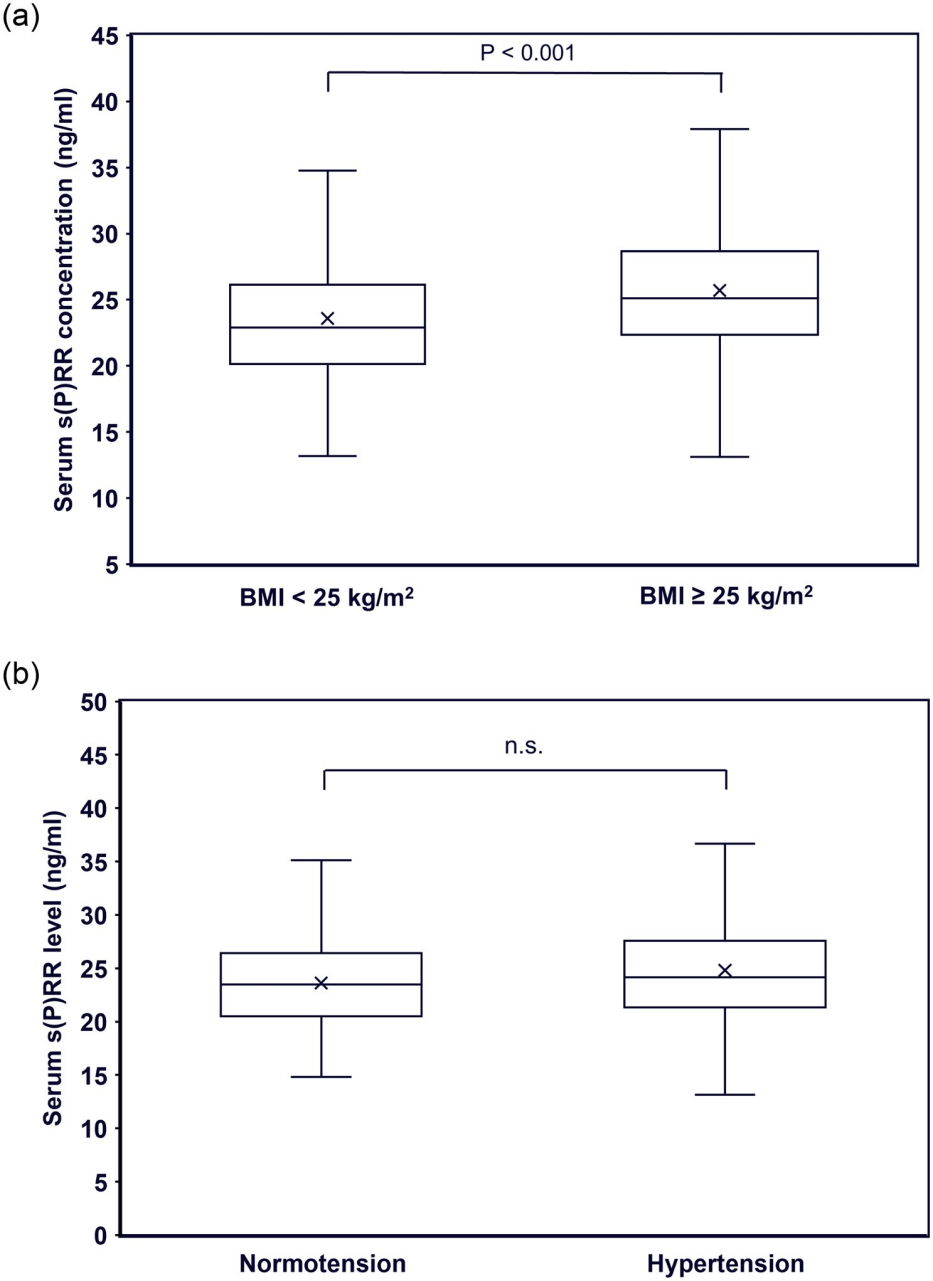
Comparisons of serum s(P)RR concentration between patients with and without obesity (a) and those with and without hypertension (b). s(P)RR, soluble (pro)renin receptor; BMI, body mass index.

**Fig 5:**
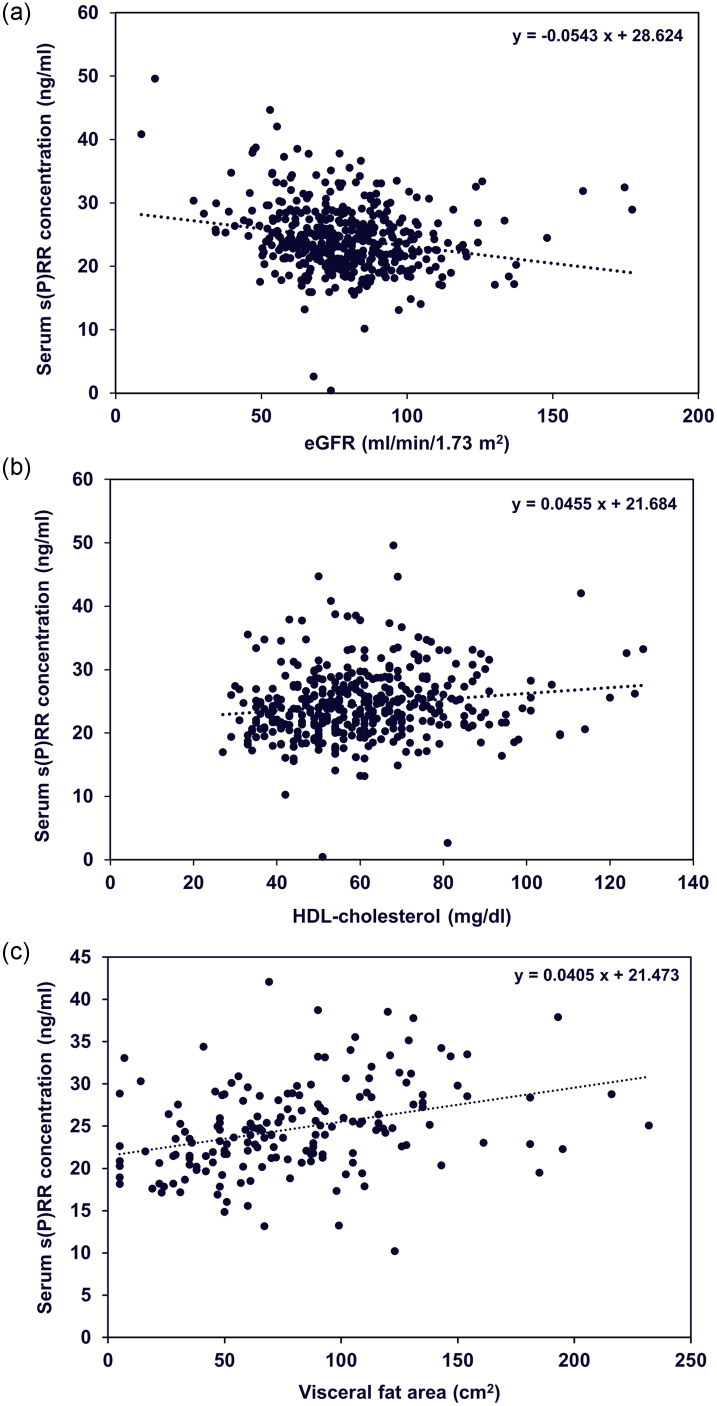
Scattergrams showing relationship between eGFR (a), HDL-cholesterol (b), or visceral fat area (c) and serum s(P)RR concentration. s(P)RR, soluble (pro)renin receptor; eGFR, estimated glomerular filtration rate; HDL, high-density lipoprotein.

Because serum s(P)RR showed a significant negative relationship with urinary s(P)RR excretion level, we also investigated the relationships between serum s(P)RR concentration and background factors. Serum s(P)RR concentration was significantly higher in patients with a BMI ≥ 25 kg/m^2^ compared to those with a BMI < 25 kg/m^2^ (Fig 4a); however, it was not significantly different between normotensive and hypertensive patients (Fig 4b). Single correlation analyses showed that age, BMI, WC, VFA, urine Alb excretion, Hb, uric acid, Cre, Ca, BS, insulin, HbA1c, TG, and hsCRP were significantly and positively correlated, and urine pH, eGFR, and HDL-cholesterol were significantly and negatively correlated with serum s(P)RR concentration (Table 4, Fig 5a–5c).

**Table 4:** Single correlation analyses with serum soluble (pro)renin receptor levels.

Variables	ρ	P
Age	0.150	0.006
BMI	0.248	<0.001
Waist circumference	0.329	<0.001
Visceral fat area	0.395	<0.001
Office BP and		
Systolic BP	0.097	0.055
Diastolic BP	-0.104	0.838
Pulse rate	0.008	0.142
Urine tests		
pH	-0.147	0.003
Albumin	0.230	<0.001
Blood tests		
Hemoglobin	0.114	0.018
Uric acid	0.306	<0.001
Creatinine	0.253	<0.001
eGFR	-0.224	<0.001
Corrected calcium	0.211	<0.001
Inorganic phosphorus	0.049	0.361
Albumin	-0.006	0.914
Intact-PTH	0.005	0.944
Blood sugar	0.189	<0.001
Insulin	0.293	<0.001
Hemoglobin A1c	0.188	<0.001
LDL-cholesterol	0.063	0.190
HDL-cholesterol	-0.304	<0.001
Triglyceride	0.365	<0.001
hs CRP	0.297	<0.001

Abbreviations: BMI, body mass index; BP, blood pressure; eGFR, estimated glomerular filtration rate; PTH, parathyroid hormone; LDL-C, low-density lipoprotein cholesterol; HDL-C, high-density lipoprotein cholesterol; hsCRP, high-sensitivity C-reactive protein; BNP, brain natriuretic peptide.

#### Multiple regression analyses with serum s(P)RR levels

Several studies have indicated that blood s(P)RR levels are significantly and negatively associated with eGFR [15, 21, 22]. Data from the present study confirmed this relationship (Table 4, Fig 5a). To compare the strength of the relationship between serum s(P)RR level and urinary s(P)RR excretion and eGFR, multiple regression analysis testing with urinary s(P)RR and eGFR as independent variables was performed. Only eGFR was selected as a possible independent variable; it was found to be significantly and negatively associated with serum s(P)RR level, suggesting that the association between serum s(P)RR level and urinary s(P)RR excretion is confounded by renal function (Table 3b).

### Study 2

**Fig 6:**
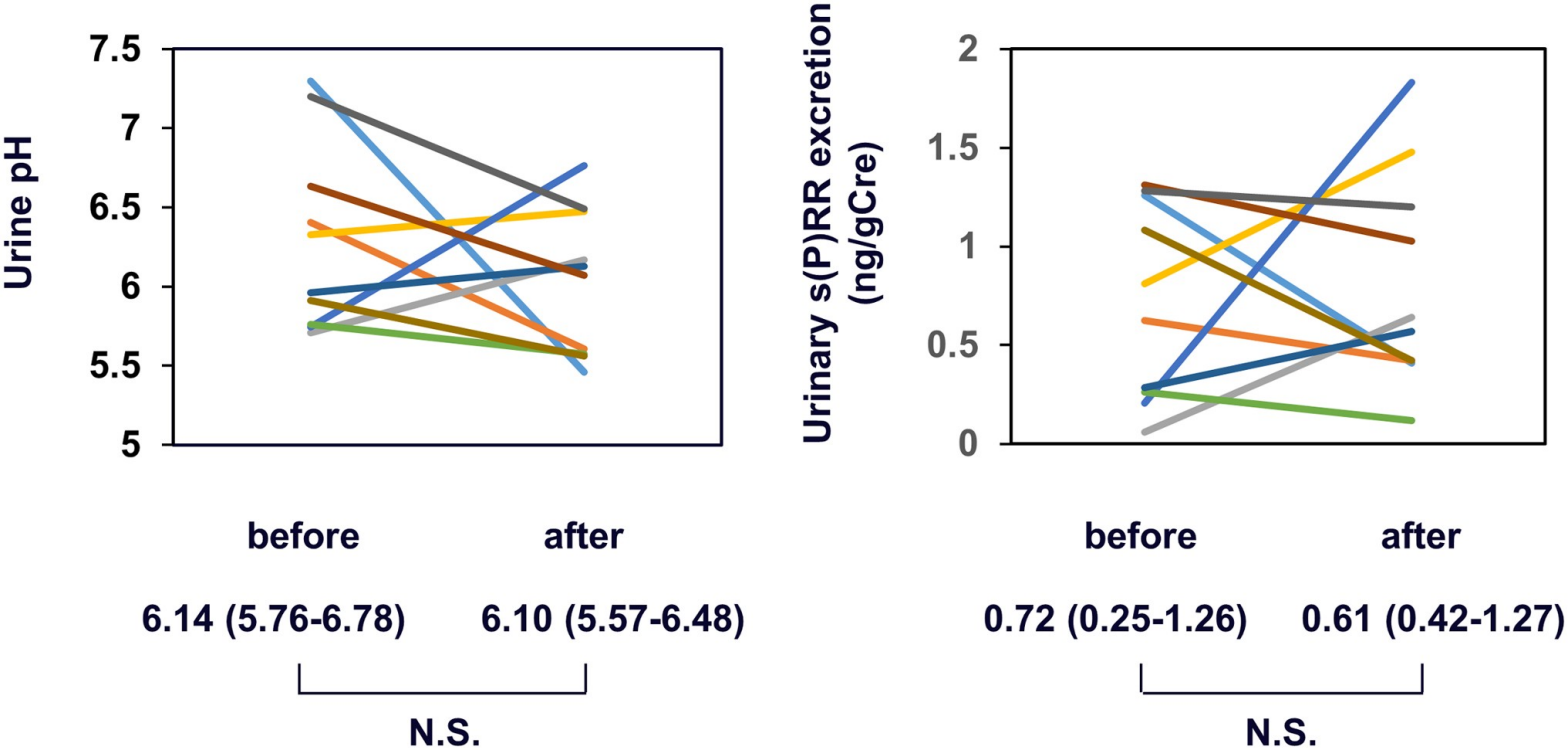
Changes in urine pH or urinary s(P)RR excretion by vitamin C treatment. The same color was used for each of the 10 volunteers. N.S., not significant. s(P)RR, soluble (pro)renin receptor.

**Fig 7:**
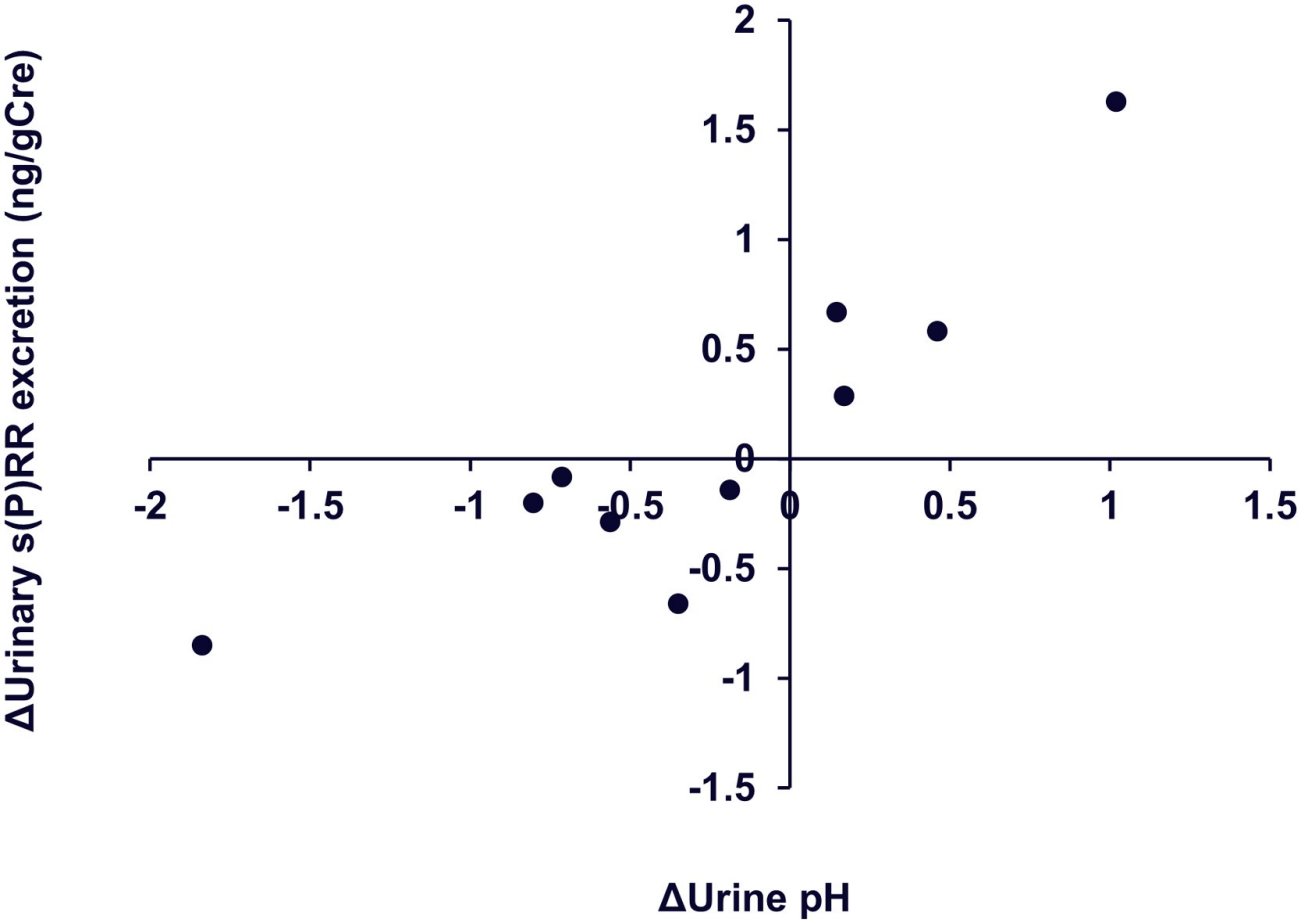
Scattergrams showing relationship between changes (Δ) in urine pH and urinary s(P)RR excretion by vitamin C treatment. Closed circles, each with 10 volunteers (r = 0.878, P < 0.05), using Spearman’s rank correlation coefficient. s(P)RR, soluble (pro)renin receptor.

The data of the study participants at enrollment are shown in Table 5. Urine qualitative tests revealed that all participants had negative urine protein, sugar, and occult blood levels. All these data confirmed their suitability as healthy volunteers. Although vitamin C was administered to the participants, unexpectedly, neither urine pH nor urinary s(P)RR excretion was significantly changed by the treatment (Fig 6), suggesting the limitation of vitamin C in reducing urine pH. However, changes in urinary s(P)RR excretion by vitamin C treatment showed significantly positive relationships with changes in urine pH (Fig 7, ρ = 0.8182, p = 0.0038).

**Table 5:** Characteristics of the study subjects.

Age (y.o.)	22±2
BMI (kg/m^2^)	23.1±1.2
Office BP and pulse rate	
Systolic BP (mmHg)	115±11
Diastolic BP (mmHg)	68±6
Pulse rate (beats/min)	59±7
Urine tests	
pH	6.14 (5.76–6.78)
s(P)RR/creatinine (ng/g creatinine)	0.72 (0.25–1.26)
Blood tests	
Creatinine (mg/dl)	0.85±0.06
eGFR (ml/min/1.73 m^2^)	96.3±7.5
Blood sugar (mg/dl)	95±4
Hemoglobin A1c (%)	5.4 (5.1–5.6)

Data are presented as mean ± standard deviation, median (interquartile range).

Abbreviations: BMI, body mass index; BP, blood pressure; s(P)RR, soluble.

(pro)renin receptor; eGFR, estimated glomerular filtration rate.

## Discussion

This is the first study to investigate the characteristics of urinary s(P)RR excretion; it demonstrated two major findings regarding urinary s(P)RR excretion in humans. First, s(P)RR could be detected in urine, and urinary s(P)RR excretion was significantly and positively correlated with urine pH, independent of other factors in humans. Second, changes in urinary s(P)RR excretion were positively correlated with changes in urine pH. These data support the notion that (P)RR expression in the kidney might play a role in urine pH regulation.

In the present study, s(P)RR was detected in urine by western blotting (Fig 1), and urinary s(P)RR concentration was measured by sandwich ELISA (Table 1). Urine pH and indices of obesity, lipid metabolism, glucose metabolism, renal function, and inflammation were correlated with urinary s(P)RR excretion (Table 2). Among the factors associated with urinary s(P)RR excretion, urine pH showed the strongest relationship with urinary s(P)RR excretion independent of other factors. These data suggest that urinary s(P)RR excretion is specifically associated with urine pH.

Interestingly, there was a significant negative correlation between serum s(P)RR concentration and urinary s(P)RR excretion (Table 2). Therefore, it is unlikely that the main source of urinary s(P)RR is blood s(P)RR filtered by the renal glomeruli, although the molecular weight of s(P)RR is 28 kDa [18] and s(P)RR could be partially filtered by the glomeruli.

The (P)RR was identified as an accessory protein of V-ATPase, which is a multimeric protein complex with a V1 sector (eight subunits) that inherits ATPase function and a V0 sector (six subunits) that mediates proton translocation driven by ATP cleavage [23, 24]. V-ATPase controls the pH of the extracellular space as well as that of intracellular compartments, depending on its actual localization (e.g., cell membranes, lysosomal membranes, membranes of secretory granules, etc.) [25]. In the kidney, (P)RR is predominantly expressed in collecting ducts and in the distal nephron; in addition it colocalizes with V-ATPase in type-A intercalated cells [26], which expels protons into the tubular lumen, thereby regulating the final urinary acidification [27]. Therefore, (P)RR may play a role in the excretion of protons into the tubular lumen via the function of V-ATPase [26]. In addition, both angiotensin II and aldosterone have been shown to directly and indirectly influence transport pathways involved in acid secretion, such as Na^+^/H^+^ exchangers and V-ATPases in different nephron segments, including the acid-secretary type-A intercalated cells in the collecting ducts [28, 29].

**Fig 8:**
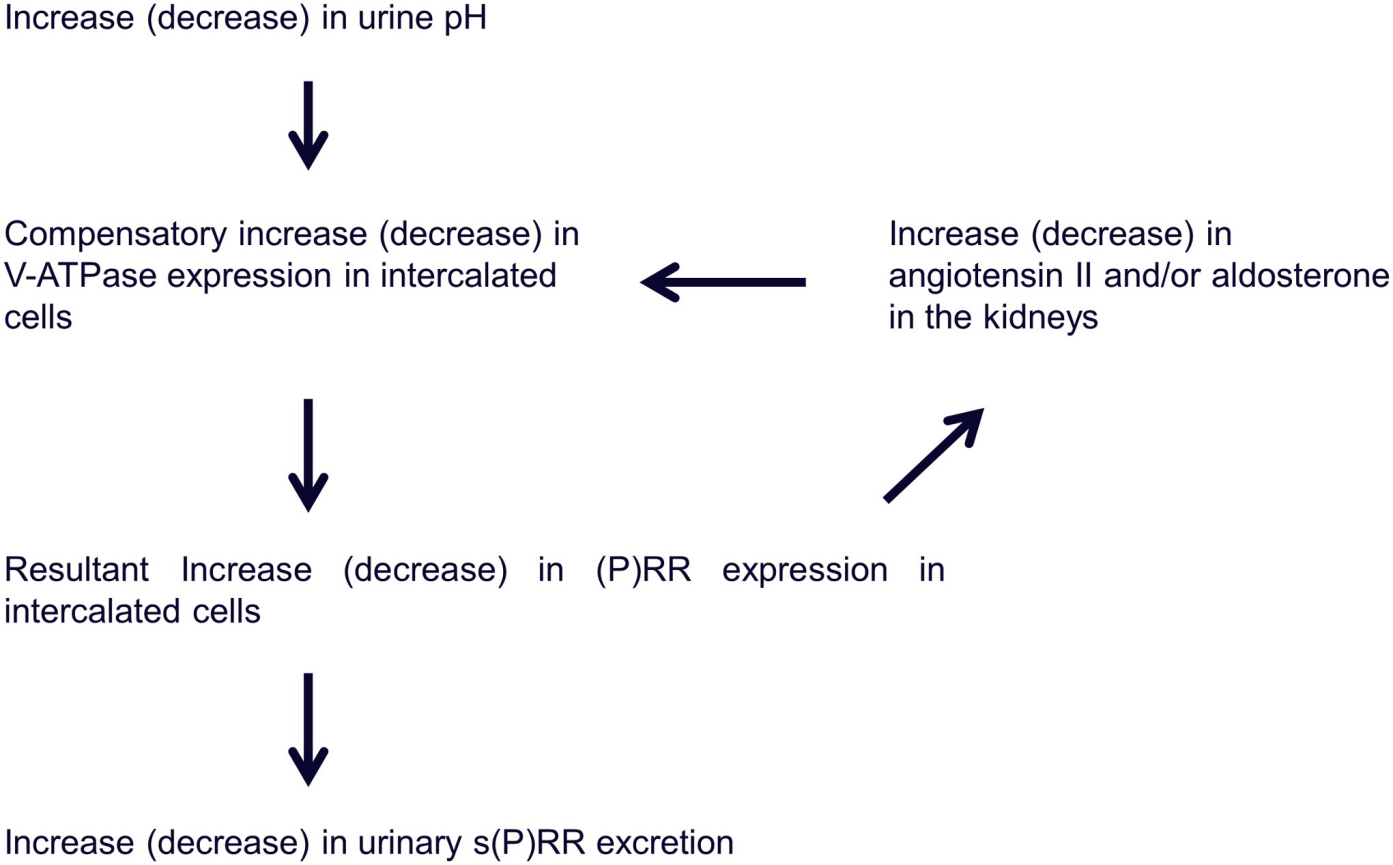
Hypothetical mechanism for the regulation of pH by (P)RR in kidney cells. V-ATPase, vacuolar H^+^-ATPase; (P)RR, (pro)renin receptor; s(P)RR, soluble (pro)renin receptor.

In the present study, to determine the causal relationship between urinary s(P)RR excretion and urine pH, we investigated the effects of vitamin C treatment, which may decrease urine pH, on urinary s(P)RR excretion levels in healthy volunteers. Changes in urine pH due to vitamin C varied among the participants, and urine pH did not change significantly in the whole group (Fig 6); however, the change in urine pH was significantly and positively associated with changes in urinary s(P)RR excretion levels (Fig 7). (P)RR is an accessory V-ATPase [3]. These data prompted us to hypothesize that urinary s(P)RR excretion is increased (or decreased) due to increased (or decreased) (P)RR expression in intercalated cells, resulting from increased (or decreased) V-ATPase activity in these cells to compensate for the increased (or decreased) urine pH (Fig 8), although this hypothesis needs to be investigated further. In addition, the possibility that (P)RR in the tubules or urinary s(P)RR causes increased secretion or decreased absorption of protons in the tubules cannot be ruled out at this moment.

Serum s(P)RR concentration showed completely different features compared with urinary s(P)RR excretion, and showed significantly positive relationships with age, BMI, WC, VFA, urine Alb excretion, Hb, uric acid, Cre, Ca, BS, insulin, HbA1c, TG, and hsCRP, and significantly negative relationships with urine pH, eGFR, and HDL-cholesterol (Table 4). These data are in accordance with those of previous investigations reporting that blood s(P)RR levels are associated with obesity [30], dyslipidemia [15], renal dysfunction [15, 21], and inflammation [22]. These findings suggest that blood s(P)RR concentration is affected by the expression levels of tissue RAS and/or tissue (P)RR in various organs. Urinary s(P)RR excretion and blood s(P)RR concentration may reflect different physiological and/or pathophysiological conditions in humans. Interestingly, urinary s(P)RR excretion was significantly negatively associated with serum s(P)RR concentration (Table 2). The reason for this association is undetermined, but it is possible that serum s(P)RR concentration is increased due to decreased renal function [15, 21] when s(P)RR production causes urinary s(P)RR excretion to be reduced. This hypothesis needs to be tested in future studies.

## Limitations

We acknowledge that this study has some limitations. First, we could not investigate the effect of urinary alkalinization agents on volunteers for ethical reasons. The causal relationship between urinary s(P)RR excretion and urinary proton excretion remains speculative. Second, we could not determine the source of s(P)RR in urine in this clinical study. Third, whether urine s(P)RR excretion can be used as a biomarker to indicate the pathological status is unclear. Further studies are required to clarify the significance of measuring urinary s(P)RR excretion.

## Conclusions

In this study, in which the characteristics of urinary s(P)RR excretion were investigated for the first time, s(P)RR could be detected in urine, and urinary s(P)RR excretion was shown to be significantly positively correlated with urine pH, independent of other factors. Alterations in urine pH caused changes in urinary s(P)RR excretion. These data suggest that (P)RR expression in the kidney might play a role in the regulation of urine pH. Further investigations are required to determine the roles of (P)RR in the regulation of urine pH in more detail as well as the usefulness of measuring urinary s(P)RR excretion in clinical settings.

## Supporting information

S1 Data (XLSX)

S2 Data (XLSX)

S1 Fig (TIF)
