# Supplementary figures and images for: Urinary soluble (pro)renin receptor excretion is associated with urine pH in humans
Source: PLoS One. 2021 Jul 26;16(7):e0254688. doi: 10.1371/journal.pone.0254688 (PMC8312976; doi:10.1371/journal.pone.0254688)

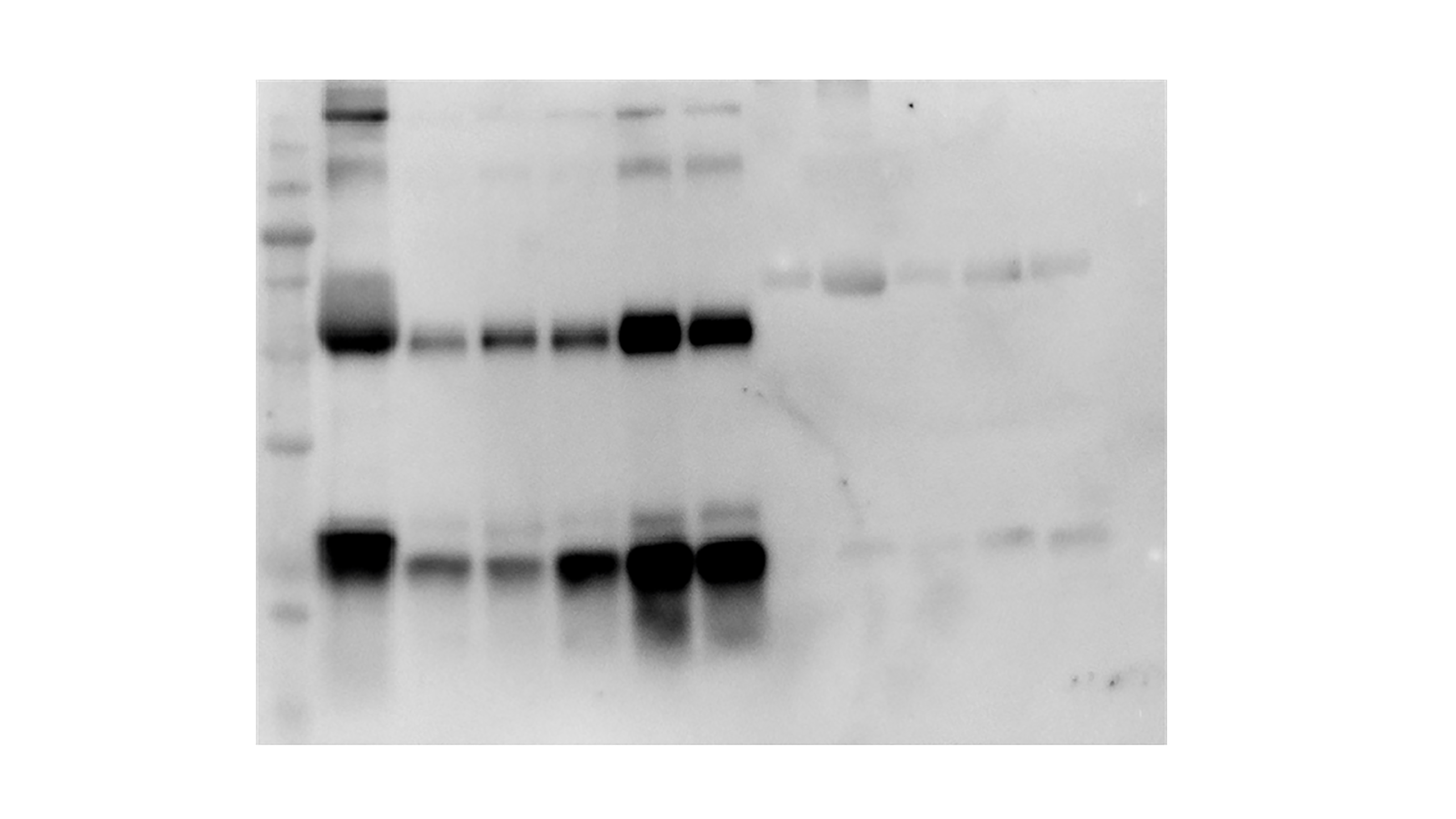

Supplement: S1 Fig — (TIF) [file pone.0254688.s003.tif]
